# Pulmonary Endometriosis: A Systematic Review

**DOI:** 10.3390/jpm14111085

**Published:** 2024-10-31

**Authors:** Konstantinos Nikolettos, Alexandros Patsouras, Sonia Kotanidou, Nikolaos Garmpis, Iason Psilopatis, Anna Garmpi, Eleni I. Effraimidou, Angelos Daniilidis, Dimitrios Dimitroulis, Nikos Nikolettos, Panagiotis Tsikouras, Angeliki Gerede, Dimitrios Papoutsas, Emmanuel Kontomanolis, Christos Damaskos

**Affiliations:** 1 Department of Obstetrics and Gynecology, University Hospital of Alexandroupolis, Democritus University of Thrace, Dragana, 68110 Alexandroupolis, Greece; k.nikolettos@yahoo.gr (K.N.); kotanidou.so@gmail.com (S.K.); nnikolet@med.duth.gr (N.N.); ptsikour@med.duth.gr (P.T.); agerede@med.duth.gr (A.G.); ekontoma@med.duth.gr (E.K.); 2 Second Department of Pulmonology, Sotiria General Hospital, 11527 Athens, Greece; patsouras.alexandros@gmail.com; 3 Department of Surgery, Sotiria General Hospital, 11527 Athens, Greece; nikosg22@hotmail.com (N.G.); papdr@yahoo.com (D.P.); 4 N.S. Christeas Laboratory of Experimental Surgery and Surgical Research, Medical School, National and Kapodistrian University of Athens, 11527 Athens, Greece; 5 Department of Obstetrics and Gynecology, University Erlangen Hospital, 91054 Erlangen, Germany; 6 First Department of Propedeutic Internal Medicine, Laiko General Hospital, Medical School, National and Kapodistrian University of Athens, 11527 Athens, Greece; annagar@windowslive.com; 7 First Surgical Department, University Hospital of Alexandroupolis, Democritus University of Thrace, Dragana, 68110 Alexandroupolis, Greece; eeffraem@med.duth.gr; 8 First University Department in Obstetrics and Gynecology, Papageorgiou General Hospital, School of Medicine, Aristotle University of Thessaloniki, 56429 Thessaloniki, Greece; angedan@auth.gr; 9 Second Department of Propedeutic Surgery, Laiko General Hospital, Medical School, National and Kapodistrian University of Athens, 11527 Athens, Greece; dimitroulisdimitrios@yahoo.com; 10 Department of Emergency Surgery, Laiko General Hospital, 11527 Athens, Greece

**Keywords:** endometriosis, thoracic, pulmonary, extra-pelvic, catamenial, syndrome, TES

## Abstract

**Background/Objectives**: Endometriosis is characterized by the presence of ectopic endometrial-like glands and stroma outside the endometrial cavity, which mainly occurs in the pelvic cavity. Pulmonary endometriosis, or thoracic endometriosis syndrome (TES), describes the rare presence of endometrial-like cells in the thoracic cavity and includes catamenial pneumothorax, catamenial hemothorax, hemoptysis, and lung nodules. Our aim is to summarize the results of all reported cases of TES. **Methods**: Extensive research was conducted through MEDLINE/PUBMED using the keywords “thoracic endometriosis”, “thoracic endometriosis syndrome”, “catamenial pneumothorax”, “catamenial hemoptysis”, and “TES”. Following PRISMA guidelines, all published cases of TES between January 1950 and March 2024 were included. A systematic review of 202 studies in English, including 592 patients, was performed. **Results**: The median age of women with TES is 33.8 years old. The most common clinical presentation is catamenial pneumothorax (68.4%), while lesions are mainly found in the right lung unilaterally (79.9%). Chest computed tomography (CT) was used alone or after an X-ray to determine the pathological findings. Ground-glass opacity nodules and cystic lesions represent the most common finding in CT, while pneumothorax is the most common finding in X-rays. Video-assisted thoracoscopic surgery (VATS) is the main therapeutic approach, usually in combination with hormonal therapy, including GnRH analogues, progestins, androgens, or combined oral contraceptives. Hormonal therapy was also administered as monotherapy. Symptom recurrence was reported in 10.1% of all cases after the treatment. **Conclusions**: High clinical awareness and a multidisciplinary approach are necessary for the best clinical outcome for TES patients. More studies are required to extract safer conclusions.

## 1. Introduction

Endometriosis is a chronic inflammatory process which affects approximately 10% of women of reproductive age [1]. It is defined by the presence of both endometrial-like stroma and glands outside the endometrial cavity [2]. Most lesions are located in the pelvis or the abdomen, and thus mainly cause pelvic pain, infertility, and uterine bleeding [1].

Thoracic endometriosis syndrome (TES) is a less frequent clinical entity. Due to the diverse symptoms of TES the exact incidence is challenging to establish. Studies suggest that 1.5% of the population have thoracic involvement of endometriosis [1]. The diaphragm, pleural cavity, and pulmonary parenchyma are affected by these lesions. TES is defined by four clinical entities, which include catamenial hemothorax (CHt), catamenial hemoptysis (CH), catamenial pneumothorax (CP), and lung nodules [3]. CH constitutes a rare entity which is characterized by recurrent hemoptysis during menstruation. The presence of endometrial tissue in the lung parenchyma or bronchus is regarded as the primary cause of this symptom. The pathophysiological mechanisms of intrabronchial or parenchymatic endometrial lesions still remain unclear. Additionally, there is no agreement on the characteristic clinical features or the ideal therapeutic alternatives for patients with lung endometriosis [3].

The aim of the current study is to present the total number of case reports presented in the international bibliography of thoracic endometriosis, and to evaluate their epidemiological features, symptoms, diagnostic methods, treatment procedures, and outcomes.

## 2. Materials and Methods

A review of the literature was conducted using the PubMed database to identify articles of case reports and case series of thoracic endometriosis. This systematic review was created in accordance with the guidelines of the Preferred Reporting Items for Systematic Reviews and Meta-Analyses (PRISMA guidelines). All the articles published between January 1950 and March 2024 that met the inclusion criteria were accumulated in this systematic review. Specifically, our research was performed using keywords, separately and in various combinations, such as lung, pulmonary, thoracic endometriosis, TES, catamenial, hemothorax, hemoptysis, and pneumothorax. This study has not been registered in any public registry.

This systematic review includes patients diagnosed with thoracic endometriosis based on clinical or pathological findings, without specific consideration of the diagnostic methods used. Data from each publication on thoracic endometriosis were extracted and summarized based on the symptoms, the imaging methods utilized and their findings, smoking status, history of pelvic endometriosis, prior gynecological procedures, therapeutic approaches, and any reported recurrences.

The initial research using the aforementioned keywords identified 895 articles. After the removal of duplicates, 885 remained, and by filtering by case reports and case series, 444 studies remained. Furthermore, we checked the references from all articles found, aiming to include any other eligible studies. The remaining articles were screened and 242 were excluded for various reasons, such as irrelevant topics regarding the diagnosis of the case report, insufficient data, or the type of the article (e.g., abstract or review). Publications in non-English languages were also excluded. There were 202 full-text articles assessed for eligibility and none of them were excluded.

### Risk of Bias

To minimize the bias risk of the systematic review of case reports and series, we predefined the inclusion and exclusion criteria as outlined previously.

Initially, the bibliography was assembled based on title and abstract by one author, and the full text screening was performed by another author. Two different authors performed the data extraction from the articles that met the inclusion criteria. Data about the age, symptoms, diagnostic methods, radiological findings, history of pelvic endometriosis and surgical history, treatment procedures, and recurrency rates in each article included were exported to an MS Excel spreadsheet to collect the same information. Two independent reviewers performed double data extraction to reduce the risk of bias assessments, and any disagreements were resolved by a third reviewer.

Some of the limitations manifested in this review are the exclusion of non-English language articles and the inconsistency of some common points among the studies included.

**Figure 1:**
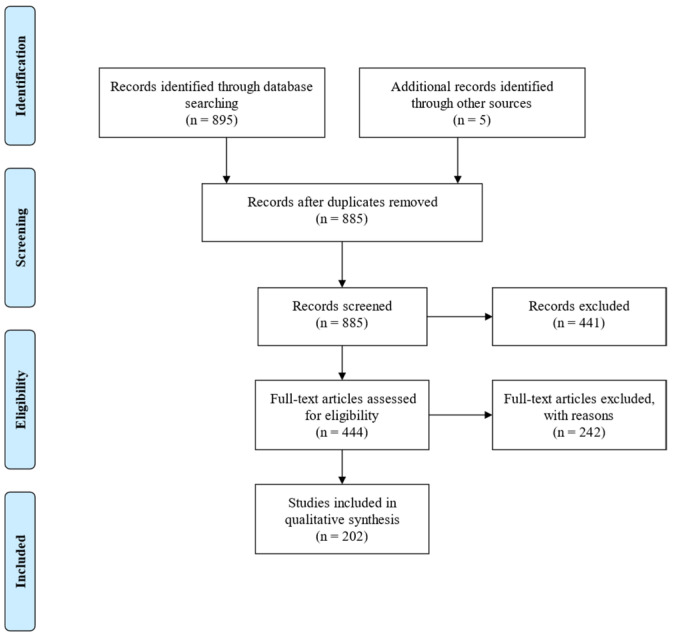
PRISMA flow diagram for the present study.

The inclusion process is shown in Figure 1 (PRISMA flow diagram).

## 3. Results

A total of 592 patients with thoracic endometriosis in 202 studies were reported and are listed below. Their main characteristics are reported in Table 1, Table 2, Table 3, Table 4, Table 5, Table 6 and Table 7. Their median age was 33.8 years old at presentation, and the most common symptom was pneumothorax (68.4%, 405/592), followed by chest pain (22%, 130/592), dyspnea (20.9%, 124/592), and hemoptysis (14.2%, 84/592). Cough as the main symptom was reported in 6.8% (40/592) of cases and pelvic pain, as a unique symptom, was presented in 2.4% (14/592). Other rare presentations were flu-like symptoms (4/592), shock state (1/592), or absence of symptoms (2/592). The presentation of the symptoms related to thoracic endometriosis is illustrated in Table 1.

In total, 35.3% of the included patients (47/133) were smokers, whereas 6% (8/133) were ex-smokers, and 58.6% (78/133) did not exhibit smoking habits. There is no information concerning smoking in the remaining 460 cases (77.5% of the total patients) (Table 2).

The location of each lesion was determined by radiological, endoscopic, or pathological methods. Most of the lesions (473/592, 79.9%) were found on the right side unilaterally, and specific ectopic endometrial lesions were encountered in 7.6% of the patients in the right lower lobe (RLL) (36/473), in 9.5% of them in the right upper lobe (RUL) (45/473), and in 2.7% in the right middle lobe (RML) (13/473) when mentioned. The lesions, which were only left-sided, were found in 7.9% of the patients (47/592). Lesions in the left upper lobe (LUL), including the lingula, and in the left lower lobe (LLL), were found in 59.6% (28/47) and in 34% (16/47) of patients, respectively. Bilateral lesions were found in 4.9% (29/592). In four patients, the lesions were observed in the trachea [129,130,131,194], whereas in another one, the location was not stated [109]. Extra-thoracic endometriosis was only detected in 47.8% of the cases (222/464). Table 3 and Table 4 summarize the location of the lesions.

On the contrary, 52.2% (242/464) of patients did not show any signs of extra-thoracic endometriosis, and in 21.5% of the total number of patients (127/592), no statement was made in the article (Table 5). The diagnostic tool (transvaginal ultrasonography, MRI, or exploratory laparoscopy) used to identify extra-thoracic endometriosis is not specified for each case.

As far as gynecological history is concerned, almost the half of the patients that mentioned their surgical history (53.5%, 212/396) had previously undergone gynecological procedures, including abortions (13.2%, 28/212), diagnostic laparoscopies (23.7%, 50/211), hysterectomies with or without oophorectomies (16.4%, 18/110), and cesarian sections (8.5%, 18/211). Many patients (31.1%, 184/592) had no history of any gynecological surgery in the past, while for the rest, 33.3% (197/592) of the patients, there is no information about their surgical history. Additional information is provided in Table 6 and Table 7.

The imaging methods used for diagnosis were chest X-ray, chest Computer Tomography (CT), chest Magnetic Resonance Imaging (MRI), and bronchoscopy. Chest X-rays were initially used in 189 out of 592 patients (31.9%). The most frequent finding in X-rays was pneumothorax (38%, 72/189), followed by normal findings (20.1%, 38/189), pleural effusion (13.8%, 26/189), consolidation (12.2%, 23/189), and nodules (9%, 17/189). Chest CT scans were most frequently conducted upon clinical symptomatology and at the beginning of the menstrual cycle (32.9%, 195/592). Ground-glass opacities (GGOs) were the most common radiological findings (21.5%, 42/195). Nodules and consolidation were found in 17.9% (35/195) and 10.8% (21/195), respectively. Cystic lesions including cavitary lesions and bullae were found in 15.4% (30/195). Pneumothorax was not so frequently encountered (10.3%, 20/195). Other rare findings included peribronchial infiltrates (2.1%, 4/195), normal findings (5.6%, 11/195), and ring-shaped lesions in only one patient. An absence of pathological findings and disappearance or reduction in the size of the lesions was frequently found in the middle of the menses. MRI and Positron Emission Tomography (PET) were only used in 14 (2.4%) [4,5,6,72,73,74,75,76,77,78,79,110,132,133] and 4 (0.7%) [7,134,135,186] cases, respectively, in order to confirm the exact position and nature of the lesions. Furthermore, bronchoscopies during symptoms were used for diagnosis in 66 out of 592 cases (11.1%). Hemorrhage or hyperemia was observed in 40.9% (27/66) of cases, whereas normal findings were noticed in 42.4% (28/66). Macroscopic lesions were noticed in 11 cases (16.7%), which constituted endobronchial endometriosis. The bronchoscopical lesions disappeared or diminished in size in 14 cases [136,137,138,139,140,187,194,195]. Finally, bronchial angiography was used in five cases (0.8%) in order to detect the site of the hemorrhage [109,111,130,141,142]. The main radiological findings are reported in Table 8, Table 9 and Table 10.

Diagnosis of endometriosis was either clinical or pathological. In total, 24.3% (144/592) of cases were histopathologically diagnosed by the presence of endometrial cells or glands. Another pathological finding was the simultaneous presence of endometrial glands, stroma, and hemosiderin-laden macrophages, which occurred in 61 cases (10.3%). Antibodies against CD-10, estrogen, or progesterone receptors were used effectively to diagnose pulmonary endometriosis in 84 cases (14.2%). The pathologist’s confirmation was derived from specimens obtained after surgery or bronchoscopy. Histopathological confirmation was derived by the use of bronchoscopy, by the use of bronchial washing or lavage, in six cases (1%). The rest of the patients had a clinical diagnosis. Table 11 summarizes the aforementioned findings.

The remaining cases (42.9%, 254/592) were diagnosed based on their clinical presentation with the assistance of radiographic techniques, as mentioned above. The correlation of the radiographic findings with the menses of women aids in the diagnosis of TES.

Thoracic surgical treatment was performed in 276 cases (46.6%). In total, 79.3% (219/276) of these operations were supported by video-assisted thoracoscopy (VATS), while in three cases robotic-assisted thoracoscopy (RATS) was performed. In the majority of cases, lobectomies, segmentectomies, and wedge resections were performed. In 34.4% (95/276), surgeons performed thoracotomies, whereas pleurectomy was performed in 38 patients. In 10 cases, the patients also underwent hysterectomy and bilateral oophorectomy (3.6%), and in thirty-nine cases (14.1%) diagnostic laparoscopy was performed.

Out of a total of 220 patients who received hormonal therapy in combination with the surgical treatment (40.1%), fifty-nine experienced at least one episode of recurrence (26.8%). Patients who received only hormonal therapy (45/549) had a recurrency stated at 33.3% (15/45). Nine patients reported symptoms during hormonal therapy [4,8,9,80,81,82,135,142,143]. Women experienced recurrence after cessation of the drugs as a result of side effects or a desire for pregnancy in 14 cases [12,27,38,39,44,51,52,53,54,55,142,147,153,196]. The drugs administered to the women as monotherapy or supplementary to the surgical treatment were androgens (41/283, 14.5%), progestins (41/283, 14.5%), gonadotropin-releasing hormones (GnRH), analogues (98/283, 34.6%), and contraceptives (44/283, 15.5%). Fourteen patients did not receive any treatment, whereas in eight case reports the treatment was not mentioned [116,134,168,193].

Minimally invasive techniques were used in nine cases, including three cases of endobronchial laser use, five cases of bronchial artery embolization, and one case of endometrial lesion cryoablation. Three out of five patients treated with bronchial artery embolization had a recurrence, and one out of three patients who underwent endobronchial laser use recurrences. The patient treated with cryoablation showed no recurrence [196]. Fourteen patients initially underwent conservative treatment [87,88,137,144,145,146,147,186,188]. Eight of them (57.1%) [145,147,188] presented with symptom recurrences and were treated either hormonally [188] or surgically [188], or spontaneously recovered [145,147].

Information on all therapeutic strategies is shown in Table 12 and Table 13.

## 4. Discussion

Lung endometriosis predominantly affects women between the ages of 20 and 40. In the current systematic review, 202 studies involving 592 patients were included. All the women were of reproductive age, except for two women aged 74 years old, who were receiving exogenous hormones for osteoporosis, and a woman of 51 years old, who was on hormonal treatment due to a past history of breast cancer [13,14]. Two of the patients were pregnant during the diagnosis and the presentation of symptoms of TES [15,112].

The most common symptom by far is catamenial pneumothorax. The symptoms most related to pneumothorax are considered to be catamenial chest pain and dyspnea. Two possible mechanisms seem to be responsible for the development of pneumothorax in lung endometriosis: the rupture of intra-pulmonary blebs, and alveolar damage due to the check-valve mechanism derived from intrabronchial endometriosis or bronchiolar constriction, following excessive production of prostaglandin [208].

Another frequent clinical presentation of TES is catamenial hemoptysis. The cause of hemorrhage is probably an endometriotic implant, either inside a large bronchus or in the lung parenchyma [148]. It is worth mentioning that no massive hemoptysis was reported in any case. The severity of hemoptysis is probably influenced by the biological activity and size of the lesion [188]. However, there is little knowledge on this issue.

As far as the location is concerned, the majority of the implants are right-sided. This may support the theory of the transabdominal–transdiaphragmatic migration of endometrial cells through the right paracolic gutter. This theory requires the existence of passage in the diaphragm between the abdomen and the thorax [205]. Thus, this theory might explain the development of pleural, but not lung, endometriosis [16,17]. In addition, the lower lobes of the lungs seem to be more affected, which supports the theory of microembolization as the pathophysiologic mechanism of lung endometriosis [89,188,206]. Small endometrial tissues cause microemboli in the lung capillaries [149]. This happens as larger quantities of blood circulate in the lower lobes than in the upper ones [89,188].

Previous gynecological procedures might represent a risk factor for the development of this disease [113,150,188]. The most frequent operations were abortions, hysterectomies, and diagnostic laparoscopies. This can occur as a result of the lymphogenic or hematogenic migration of endometrial cells to the pulmonary parenchyma [150]. We should also highlight that the existence of pulmonary endometriosis is not always associated with abdominal or pelvic endometriosis. However, this does not mean that patients with pulmonary endometriosis should not be checked for other endometrial sites.

Most pathological findings reported endometrial glands, stroma, and hemosideren-laden macrophages. The use of other methods, including immunohistochemistry and anti-estrogen/progesterone receptor antibodies, can be helpful for diagnosis in the case of insufficient endometrial samples. Pseudoinfiltrative patterns without cytologic atypia or microscopic foci inside the bronchovascular bundles with signs of hemorrhage can be found in parenchymal lesions [13]. Endometrial cells can also be found after bronchial washing through bronchoscopy.

A range of diagnostic tests can aid in the differential diagnosis of TES. Beneficial tools include chest radiographs (CXR), CT scans, MRI, and bronchoscopy. The first radiologic examination was usually a chest X-ray. The most common finding in chest X-rays was pneumothorax, followed by consolidation probably due to hemorrhage and lung nodules. Pneumothorax was usually associated with chest pain and dyspnea. Chest CT was the most helpful imaging method for the location of lesions. The radiological findings included ground-glass opacities, consolidation due to hemorrhage, lung nodules, cystic lesions, and pneumothorax. The disappearance of CT lesions during the menstrual cycle favours thoracic endometriosis. MRI was used successfully in order to identify the exact location of the endometrial lesions. Bronchoscopy is not a suitable procedure for distal lesions, although it can reveal sites of hemorrhage [83,132,148,151]. Visible intrabronchial endometrial sites are reported in few cases, including purplish-red or brown submucosal patches in the airways [130,136,152,194]. Even though bronchoscopy cannot histologically confirm the diagnosis, the disappearance of the previous findings in bronchoscopies occurring in the middle of the menses should raise suspicion of endometriosis [140,142,187,195]. Finally, angiography was used in certain cases in order to confirm the exact site of hemorrhaging [109,111,130,141]. The typical findings were prominent vasculature and vascular stains in the absence of endometriosis-characteristic features [109].

The treatment of thoracic endometriosis includes several approaches. Surgical resection can be either used as first-line or second-line treatment after failure or adverse effects of hormone administration [141]. Video-assisted thoracoscopy and surgery are currently mostly used for the resection of endometrial lesions [18,72,114,153,154]. These include wedge resections and lobectomies. Exploratory thoracotomies and open surgeries can occur when VATS is not effective [19,197]. Surgeries can vary from parenchymal sparing to lobectomies [141,148]. Chest-tube placement is used in cases with life-threatening pneumothorax [17]. Minimally invasive, non-pharmacological treatments with satisfactory clinical outcomes are also reported in the literature. These include bronchoscopical ND-YAG laser treatment and cryoablation through CT guidance [89,152,196]. Bronchial artery embolization failed to treat the patient permanently [89]. The most effective treatment is hysterectomy with bilateral salpigo-oophorectomy [111,134,155]. However, this therapeutic approach should be limited in certain cases due to the multiple side effects of artificial menopause. However, there is a chance of recurrence if hormonal replacement treatment is administrated or partial oophorectomy has been conducted [3,13,148].

Hormone treatment has been used for the management of thoracic endometriosis. The aim of these drugs is to block the ovarian stimulation of the ectopic endometrial tissue [208]. Drugs including oral contraceptives, GnRH agonists, progestin analogs, and androgens have been effectively used for this purpose, as mentioned above. To date, no specific superiority of one drug over the others has been demonstrated, neither in terms of efficacy nor side effects. The drugs mostly used are GnRH analogues and combined oral contraceptives. GnRH agonists cause hypogonadotropic hypogonadism, since they reduce luteinizing and follicle-stimulating hormone [113]. Their side effects are a result of low levels of estrogen and progesterone. Oral combined contraceptives inhibit the secretion of GnRH, follicle-stimulating hormone (FSH), and luteinizing hormone [LH]. An androgen frequently used, danazol, leads to anovulation through an increase in blood testosterone and an inhibition of steroidogenesis, but might cause androgenetic side effects or liver damage [207]. Progestins influence the release of GnRH, leading to a decrease in the secretion of FSH and LH [209]. Hypoestrogenism causes the atrophy of ectopic endometrium. The most common side effects are weight gain, hypertension, uterine bleeding, depression, and breast tenderness [141,154,198]. However, hormonal therapy alone seems to be associated with a greater number of recurrences than surgical approaches, based on the data collected by the case reports and the series included in this systematic review. The recurrences are a result of the cessation of the drug due to side effects, and usually do not occur during administration periods [73,84,130,140,141,148,151]. In some cases, a more aggressive approach has been implemented, with both surgical and hormonal treatments (pro- or neo-adjuvant), in order to prevent recurrences and offer the best clinical outcome [18,114,154].

The administration of hormonal drugs renders pregnancy impossible and is associated with various side effects, as mentioned above [141]. Surgery can also have a variety of complications. Furthermore, some support that ectopic endometrial cells do not surely show similar behaviour to normal endometrial tissue towards hormonal influence [147]. No report for massive hemoptysis has ever been published. Thus, conservative treatment has been used as therapeutic approach in this disease. It has been demonstrated that women with mild symptoms show no recurrence of their symptoms without any therapeutic intervention [137,144]. Conservative treatment and follow-up can be a therapeutic approach for these women [147,188]. The ESHRE guidelines recommend the proposal of hormonal treatment in these cases, but if surgery is indicated, the involvement of a thoracic surgeon and/or other relevant specialists is suggested [2].

This review has some limitations: Articles in non-English languages were not included in this study. The use of case series and case reports provides knowledge about this disease. However, these articles do not share many common data points that can be used to extract safe conclusions concerning incidence or cause–effect. Finally, the follow-up periods are short in many studies.

## 5. Conclusions

It is obvious that pulmonary endometriosis is more frequent than thought. Its main clinical characteristic is catamenial hemoptysis, which coincides with the menstrual cycle of the woman. The radiological disappearance of the lesion in the middle of the woman’s cycle is another important point to mention. However, no specific guidelines exist concerning the diagnosis and therapeutic management of these patients. Thus, its prompt management requires a high level of clinical expertise and a multi-disciplinary approach. Finally, it is of paramount importance to create an international registry of patients with clear and common data points, in order to facilitate data extraction and acquire better knowledge of the disease.

## Figures and Tables

**Table 1:** Symptoms of pulmonary endometriosis mentioned in the studies included in the systematic review.

Symptom	Number of Cases (592)	References
Pneumothorax	405	[4,5,6,7,8,9,10,11,12,13,14,15,16,17,18,19,20,21,22,23,24,25,26,27,28,29,30,31,32,33,34,35,36,37,38,39,40,41,42,43,44,45,46,47,48,49,50,51,52,53,54,55,56,57,58,59,60,61,62,63,64,65,66,67,68,69,70,71]
Chest pain	130	[3,5,6,8,9,10,11,12,13,14,15,16,17,18,19,20,21,23,25,26,27,28,29,32,35,36,38,40,42,43,44,45,46,50,51,52,53,54,55,56,58,59,62,64,66,68,69,70,72,73,74,75,76,77,78,79,80,81,82,83,84,85,86,87,88,89,90,91,92,93,94,95,96,97,98,99,100,101,102,103,104,105,106,107,108]
Dyspnea	124	[6,7,8,9,10,11,13,14,15,17,21,23,25,27,36,39,42,43,44,47,51,52,56,59,60,63,64,66,68,69,70,72,76,79,80,82,86,87,91,93,95,96,97,98,99,100,103,106,108,109,110,111,112,113,114,115,116,117,118,119,120,121,122,123,124,125,126,127,128]
Catamenial hemoptysis	84	[3,4,10,13,19,35,37,58,73,81,82,83,84,85,88,89,90,129,130,131,132,133,134,135,136,137,138,139,140,141,142,143,144,145,146,147,148,149,150,151,152,153,154,155,156,157,158,159,160,161,162,163,164,165,166,167,168,169,170,171,172,173,174,175,176,177,178,179,180,181]
Cough	40	[9,13,16,39,44,47,52,61,63,73,82,86,87,92,99,100,106,108,109,117,119,120,125,127,134,156,181]
Hemothorax	22	[44,77,82,106,126,182,183]
Pelvic–abdominal pain	14	[5,6,41,48,75,85,105,128,177,183,184,185]
Hydropneumothorax	12	[39,46,47,63,76,82,88,100,119,122,127]
Flu-like	4	[58,65,133,186]
Shock state	1	[58]
Asymptomatic	2	[13,182]

**Table 2:** Smoking habits of the patients included in the systematic review.

Smoking Habits	Number of Cases (592)	References
Smokers	47	[67,69,70,102,107,111,137,147,164,171,186,187,188,189,190]
Ex-smokers	8	[107,133,135,147,188,191]
Non-smokers	78	[8,9,12,21,22,25,27,28,39,40,41,42,46,50,60,61,71,81,84,85,87,92,93,100,101,107,112,114,117,120,121,131,136,140,152,153,161,166,167,174,176,178,180,183,188,192]
Not mentioned	460	[4,5,6,7,10,11,13,14,15,17,18,19,20,23,24,26,29,30,31,32,33,34,35,36,37,38,43,44,45,47,48,49,51,52,53,54,55,56,57,58,59,62,63,64,65,66,68,72,73,74,75,76,78,79,80,82,83,86,88,89,90,91,94,95,96,97,98,99,103,104,105,106,108,109,110,115,116,118,119,122,123,124,125,126,127,128,129,130,132,134,138,139,141,142,143,144,145,146,148,149,150,151,154,155,156,157,158,159,160,162,163,165,168,169,170,172,173,175,177,179,181,182,184,185,190,193,194,195,196,197,198,199,200,201,202,203]

**Table 3:** Location of endometrial lesions in each lung of patients included in the systematic review.

Location	Number of Cases (592)	References
Right lung	473	[4,5,6,7,8,9,10,11,12,13,15,16,17,18,19,20,21,22,24,25,26,27,28,29,30,31,32,33,34,35,37,38,39,40,41,43,44,45,47,48,49,50,51,52,54,55,56,57,58,59,60,61,62,63,64,65,66,67,68,69,70,73,74,75,76,77,78,79,80,81,82,83,84,85,86,87,89,90,91,92,93,94,95,96,97,98,99,100,101,102,103,104,105,106,107,108,109,110,114,116,118,120,121,122,123,124,125,126,127,128,129,130,131,132,137,138,139,140,141,143,146,147,148,149,150,153,154,158,159,160,161,164,165,166,167,169,172,174,175,176,177,179,181,182,185,188,189,190,191,192,193,194,195,196,198,199,200,201,202]
Left lung	47	[4,12,13,14,23,57,72,88,90,111,116,117,133,134,136,142,144,145,151,152,162,168,170,171,173,178,180,187,189,190,191,192,194,197,200,204]
Bilateral	29	[13,36,42,46,47,57,71,82,88,90,107,109,115,116,119,135,155,156,157,184,186,189,193,196]

**Table 4:** Location of endometrial lesions in each lobe of patients included in the systematic review.

Location	Number of Cases (138)	References
Right upper lobe	45	[10,13,16,17,42,63,65,81,85,108,128,131,132,137,138,139,143,147,153,154,158,159,163,164,165,166,169,177,188,192,196,201]
Right middle lobe	13	[130,147,149,157,160,165,174,176,179,188,192,195]
Right lower lobe	36	[13,16,18,19,61,64,83,89,114,115,130,131,140,141,146,147,148,149,150,156,157,165,172,175,181,184,185,188,192,195,196,198]
Left upper lobe–Lingula	28	[13,14,23,117,131,136,142,151,152,157,168,170,171,173,192,196,197]
Light lower lobe	16	[13,72,115,133,134,144,145,152,156,162,173,178,180,184,187]

**Table 5:** Extra-thoracic endometriosis diagnosed in patients included in the systematic review.

Extra-thoracic Endometriosis	Number of Cases (592)	References
Yes	222	[5,6,7,12,13,18,21,25,28,30,31,32,33,34,35,37,40,41,42,45,46,47,48,55,56,57,59,61,62,63,64,65,66,69,70,71,72,73,74,75,77,78,79,80,82,85,86,88,90,93,95,97,98,99,100,101,102,104,105,106,107,109,110,114,115,116,118,121,122,123,126,128,134,135,158,159,166,167,183,185,189,190,192,199,200,202,204]
No	242	[3,4,9,13,20,22,23,31,32,35,38,39,43,54,57,58,59,66,76,81,82,83,86,106,107,111,117,118,125,129,130,132,136,138,140,141,142,143,144,145,147,152,155,161,170,174,176,177,180,181,183,184,186,187,189,190,192,194,196,198,199]
Not mentioned	127	[8,10,11,14,15,17,19,24,26,27,29,30,36,44,49,50,51,52,53,60,67,68,84,87,89,91,92,94,96,103,108,112,119,120,124,127,131,133,137,139,146,148,149,150,151,153,154,156,157,160,162,163,164,165,168,169,171,172,173,175,178,179,182,188,191,193,195,197,200,201,203]

**Table 6:** Previous gynecological procedures of patients included in the systematic review.

Previous Gynecological Procedures	Number of Cases (592)	References
Yes	212	[13,15,18,21,28,29,32,35,42,45,46,48,55,58,59,61,63,64,66,70,72,73,78,82,83,86,90,93,95,98,105,107,114,115,116,119,122,123,131,133,134,135,136,138,142,144,145,146,147,148,149,150,151,152,153,154,155,156,157,158,159,161,164,166,169,170,171,172,173,175,179,180,181,183,184,188,190,191,192,194,195,197,198,199,200,203,204]
No	184	[3,9,24,32,35,39,40,52,59,82,86,87,89,90,104,107,109,125,126,128,132,137,162,167,182,183,187,188,190,191,192,194,195,199,200]
Not mentioned	197	[4,5,6,7,8,10,11,12,13,14,17,19,20,22,23,25,26,27,30,31,33,34,36,37,38,41,43,44,47,49,50,51,53,54,56,57,60,62,65,66,67,68,69,71,74,75,76,77,79,80,81,84,85,88,91,92,94,96,97,99,100,101,102,103,106,108,109,110,111,112,117,118,120,121,124,127,129,130,139,140,141,143,160,163,165,168,174,176,177,178,183,185,186,189,193,196,201,202]

**Table 7:** Type of main previous gynecological procedures of patients included in the systematic review.

Previous Gynecological Procedure	Number of Cases (211)	References
Abortions	28	[15,55,64,115,133,138,142,144,145,146,150,151,154,155,164,165,169,171,172,173,179,180,181,184,194,195,197,198,203]
Dilatations and Curettages	44	[13,15,55,64,73,82,83,115,131,133,136,138,142,144,145,146,147,150,151,154,155,156,161,164,169,170,171,172,173,179,180,181,184,195,197,198]
Cesarian sections	19	[13,29,66,73,107,135,136,144,152,153,161,170,175,191,194,197]
Hysterectomy with/without oophorectomy	18	[3,13,20,37,41,45,46,77,96,116,117,135,136,148,149,192,200,204]
Diagnostic Laparotomy	11	[28,58,72,82,90,93,95,119,166,200,204]
Cystectomy	9	[35,42,48,59,66,123,148]
Myomectomy	5	[61,134,199]
Diagnostic Laparoscopy	50	[21,28,32,55,63,66,78,86,90,98,105,107,114,122,142,157,158,159,161,183,190,192,199]

**Table 8:** Chest X-ray findings of patients with pulmonary endometriosis included in the systematic review.

Chest X-Ray Findings	Number of Cases (189)	References
Pneumothorax	72	[5,6,7,8,10,13,14,15,17,18,20,21,22,23,25,26,27,28,29,32,33,36,37,38,39,40,43,46,47,48,50,51,52,54,55,56,59,60,61,62,63,64,65,66,68,69,70,72,92,118]
Nodules	17	[13,33,37,44,61,75,89,119,133,134,155,175,184,185,186]
Consolidation	23	[13,37,63,75,89,109,115,123,130,133,134,143,145,148,151,155,170,175,185,197]
Pleural effusion	26	[3,7,42,47,64,87,91,92,94,95,96,97,98,99,102,103,109,110,119,120,124,125,126,127,128,202]
Hydropneumothorax	10	[46,47,76,88,100,110,119,122,124,127]
Hemothorax	1	[77]
Atelectasis	2	[70,109]
No pathological findings	38	[73,83,84,85,93,132,135,136,137,138,139,140,141,144,146,147,151,153,154,159,161,162,165,172,173,174,176,180,181,192,194]

**Table 9:** Chest computed tomography findings of patients with pulmonary endometriosis included in the systematic review.

Chest CT ^1^ Findings	Number of Cases (195)	References
Cystic lesions (Bullae–Cavities)	30	[12,14,15,18,19,23,37,71,101,108,109,114,127,134,135,137,146,151,154,156,158,159,161,163,165,175,179,187,192,196]
Pneumothorax	20	[5,8,9,11,16,17,18,19,21,28,33,37,41,43,48,65,69,70,72,106]
Ground-glass opacities	42	[19,85,89,109,135,140,142,143,147,150,152,153,154,156,158,162,163,164,165,166,174,175,176,177,178,179,192,195]
Ring-shaped lesions	1	[115]
Nodules	35	[6,18,19,32,33,35,37,46,67,84,89,101,106,111,117,132,133,137,146,151,154,156,158,159,166,175,179,184,185,186,188,192,195]
Peribronchial infiltrates	4	[130,148,152,194]
Consolidation	21	[4,13,37,81,93,109,115,130,138,144,145,151,157,160,172,173,181,188,195,197,198]
Pleural effusion	22	[7,45,64,80,87,91,93,97,98,99,103,109,110,120,123,126,127,128,167,182,202,203]
Hydropneumothorax	7	[39,64,88,100,122,125,127]
Hemothorax	2	[17,77]
No pathological findings	11	[21,50,63,73,74,76,135,139,162,170,194]

^1^ CT: Computed tomography.

**Table 10:** Bronchoscopical findings of patients with pulmonary endometriosis included in the systematic review.

Bronchoscopical Findings	Number of Cases (66)	References
Macroscopic (endometrial) lesions	11	[4,111,117,129,130,134,136,137,152,194,198]
Hemorrhage–Hyperemia	27	[10,19,83,84,89,114,129,131,138,139,140,141,142,143,144,145,150,151,156,163,164,165,172,174,177,180,187,195,197]
No pathological findings	28	[12,19,71,77,79,80,85,131,132,142,144,146,147,159,168,170,171,172,173,193,194,195,203,205]

**Table 11:** Histopathological findings of patients with pulmonary endometriosis included in the systematic review.

Histopathological Findings	Number of Cases (295)	References
Endometrial cells or glands	144	[7,8,9,10,13,14,17,18,20,21,22,25,29,32,33,35,42,43,44,45,46,47,50,51,52,56,57,59,62,64,65,66,68,70,72,73,74,77,78,79,80,81,83,84,86,88,89,91,95,97,98,99,100,101,102,103,104,106,112,113,115,119,123,125,126,127,130,131,133,134,135,137,138,143,144,145,147,148,150,151,153,155,156,157,160,162,164,165,167,168,170,171,172,175,176,177,181,182,184,187,191,192,193,194,196,197,198,199,200,201,205,206,207]
Endometrial cells through washing or lavage	6	[130,140,145,152,177,194]
Glands, stroma, and hemosiderin-laden macrophages	61	[13,16,19,48,111,114,116,128,133,134,148,149,150,153,154,155,157,160,163,165,169,171,173,174,175,178,189,192,193,195,197,198,199]
ER ^1^, PR ^2^, CD-10 ^3^	84	[17,21,29,33,34,52,83,86,102,112,113,123,127,131,145,148,163,165,170,172,185,189,196,198,200,206,207,208]

^1^ ER: Estrogen receptor; ^2^ PR: progesterone receptor; ^3^ CD: cluster of differentiation.

**Table 12:** Therapeutic approach of patients with pulmonary endometriosis included in the systematic review.

Therapeutic Approach	Number of Cases (592)	References
**Surgical treatment**	276	
Thoracotomy	95	[9,11,27,29,33,35,43,45,50,51,53,62,69,70,72,73,74,77,83,88,92,93,96,98,99,101,119,121,133,134,135,137,140,145,147,153,156,157,159,160,161,162,165,166,168,170,172,173,176,177,179,180,181,184,189,192,197,198,200,208]
Pleurectomy	38	[17,20,31,43,48,51,60,93,100,101,117,153,159,164,166,190,197]
Hysterectomy with/without oophorectomy	10	[20,46,77,90,95,109,128]
Laparoscopy	39	[35,73,74,78,90,104,105,126,183,185,202]
Laparotomy	7	[20,41,77,102,109,121,128]
Minimally invasive techniques	231	
*Bronchial artery embolization*	*5*	*[89,168,189]*
*Endobronchial laser*	*3*	*[111,129,152]*
*Cryoablation*	*1*	*[196]*
*VATS* ^1^	*219*	*[9,11,12,14,16,18,21,22,23,25,27,28,30,31,* *32,33,34,36,37,38,40,43,45,48,49,50,51,53,* *55,57,61,64,65,66,67,70,72,76,77,90,91,96,* *99,101,105,106,107,108,109,110,114,116,* *118,122,123,124,125,127,131,132,133,138,* *143,153,154,156,161,163,165,167,173,174,* *175,176,178,179,182,184,187,188,189,190,* *191,192,195,200,201]*
*RATS* ^2^	*3*	*[7,63,79]*
**Hormonal treatment**	63	
Androgens	41	[15,47,62,81,82,83,111,118,119,127,130,136,144,148,151,170,172,180,194]
Progestins	41	[4,5,11,14,16,17,23,24,25,26,28,32,58,73,75,78,92,93,97,104,112,119,124,125,126,127,128,153,159,164,169,174,185,192]
GnRH ^3^ analogues	98	[6,9,11,20,21,22,25,26,29,30,31,46,49,51,52,53,56,58,59,63,65,69,71,74,78,92,93,95,96,99,100,101,102,105,107,109,117,119,124,128,136,137,138,142,148,150,151,153,154,158,160,161,163,165,168,170,173,184,190,192,195,197,198,207]
Combined oral contraceptives	44	[8,16,39,75,106,107,108,136,147,150,158,168,177,188,199]
**Surgical + Hormonal treatment**	220	[4,5,6,8,9,11,14,15,16,17,18,20,21,22,23,24,25,26,28,29,30,31,32,35,44,46,47,49,51,52,53,56,58,59,61,63,65,69,70,71,73,74,75,76,77,78,91,92,93,95,96,97,98,99,100,101,102,104,105,106,107,109,112,113,117,118,119,120,124,125,126,127,128,130,132,134,135,136,137,138,142,148,150,151,153,154,157,158,159,160,161,162,164,165,167,168,169,173,174,182,184,185,190,192,194,195,197,198,199,205,206,207,208]
**None**	14	[87,88,137,144,145,146,147,186,188]
**Not mentioned**	8	[116,134,168,193]

^1^ VATS: Video-assisted thoracoscopy; ^2^ RATS: Robotic-assisted thoracoscopy; ^3^ GnRH: Gonadotropin-releasing hormones.

**Table 13:** Recurrency after different therapeutic approaches in patients with pulmonary endometriosis included in the systematic review.

Therapeutic Approach	Number of Cases	References
**Surgical treatment**	4	
Minimally invasive techniques		
*Bronchial artery embolization*	*3*	*[168]*
*Endobronchial laser*	*1*	*[111]*
**Hormonal treatment**	16	[4,8,9,81,82,141,142,143,148,151,188,192]
**Surgical + Hormonal treatment**	40	[11,23,30,31,32,38,40,49,55,57,63,67,69,73,82,86,106,111,167,182,192,199,200]
**Not mentioned**	43	[6,13,19,21,24,28,36,41,48,51,53,59,66,74,87,88,92,94,100,105,107,115,116,124,126,131,134,146,147,149,158,162,168,173,178,186,193,196,202]

## Data Availability

No new data were created or analyzed in this study. Data sharing is not applicable to this article.

## References

[1] Andres M.P., Arcoverde F.V.L., Souza C.C.C., Fernandes L.F.C., Abrão M.S., Kho R.M. (2020). Extrapelvic Endometriosis: A Systematic Review. J. Minim. Invasive Gynecol..

[2] Becker C.M., Bokor A., Heikinheimo O., Horne A., Jansen F., Kiesel L., King K., Kvaskoff M., Nap A., Petersen K. (2022). ESHRE guideline: Endometriosis. Hum. Reprod. Open.

[3] Joseph J., Sahn S.A. (1996). Thoracic endometriosis syndrome: New observations from an analysis of 110 cases. Am. J. Med..

[4] Parker C.M., Nolan R., Lougheed M.D. (2007). Catamenial hemoptysis and pneumothorax in a patient with cystic fibrosis. Can. Respir. J..

[5] Härkki P., Jokinen J.J., Salo J.A., Sihvo E. (2010). Menstruation-related spontaneous pneumothorax and diaphragmatic endometriosis. Acta. Obstet. Gynecol. Scand..

[6] Lameira P., Abecasis M., Palma S., Leitão J. (2022). Catamenial pneumothorax: A rare manifestation of endometriosis. Radiol. Case Rep..

[7] Giordano T., MacDonald W. (2020). Thoracic endometriosis presenting as recurrent pleural effusions. Radiol. Case Rep..

[8] Akal M., Kara M. (2002). Nonsurgical treatment of a catamenial pneumothorax with a Gn-RH analogue. Respiration.

[9] Staring G., Monteiro F., Barracha I., Amorim R. (2021). Multi-loculated catamenial pneumothorax: A rare complication of thoracic endometriosis. Cureus.

[10] Karpel J.P., Appel D., Merav A. (1985). Pulmonary endometriosis. Lung.

[11] Hirono T., Feng Y., Wang W., Yu H. (2024). Spontaneous recurrent menstrual pneumothorax: A case report. Ann. Med. Surg..

[12] Takahashi R., Kurihara M., Mizobuchi T., Ebana H., Yamanaka S. (2017). Left-sided catamenial pneumothorax with thoracic endometriosis and bullae in the alveolar wall. Ann. Thorac. Cardiovasc. Surg..

[13] Flieder D.B., Moran C.A., Travis W.D., Koss M.N., Mark E.J. (1998). Pleuro-pulmonary endometriosis and pulmonary ectopic deciduosis: A clinicopathologic and immunohistochemical study of 10 cases with emphasis on diagnostic pitfalls. Hum. Pathol..

[14] Yukumi S., Suzuki H., Morimoto M., Shigematsu H., Sugimoto R., Sakao N., Abe M., Watanabe A., Kitazawa S., Sano Y. (2020). A case of thoracic endometriosis-related pneumothorax in a menopausal woman. Gen. Thorac. Cardiovasc. Surg..

[15] Yoshioka H., Fukui T., Mori S., Usami N., Nagasaka T., Yokoi K. (2005). Catamenial pneumothorax in a pregnant patient. Jpn. J. Thorac. Cardiovasc. Surg..

[16] Van Schil P.E., Vercauteren S.R., Vermeire P.A., Nackaerts Y.H., Van Marck E.A. (1996). Catamenial pneumothorax caused by thoracic endometriosis. Ann. Thorac. Surg..

[17] Morcos M., Alifano M., Gompel A., Regnard J.F. (2006). Life-threatening endometriosis-related hemopneumothorax. Ann. Thorac. Surg..

[18] Sakai T., Azuma Y., Sano A., Tochigi N., Iyoda A. (2020). Catamenial pneumothorax with pulmonary fistula identified during surgery. Ann. Thorac. Surg..

[19] Ucvet A., Sirzai E.Y., Yakut F.C., Yoldas B., Gursoy S. (2014). Thoracic pulmonary endometriosis: Two reports of a rare disease. Arch. Bronconeumol..

[20] Arunthari V., Sevin B.U., Krishna M., Johnson M.M. (2008). Catamenial pneumothorax with umbilical and diaphragmatic endometriosis: A case report and review of the literature. South Med. J..

[21] Albores J., Fishbein G., Bando J. (2015). A 34-year-old woman with recurrent right-sided chest pain and dyspnea. Chest.

[22] Scarnecchia E., Inzirillo F., Declich P., Della Pona C. (2020). Thoracic endometriosis-related non-catamenial pneumothorax with peculiar histological findings. Gen. Thorac. Cardiovasc. Surg..

[23] Yukumi S., Suzuki H., Morimoto M., Shigematsu H., Okazaki M., Abe M., Kitazawa S., Nakamura K., Sano Y. (2016). Two young women with left-sided pneumothorax due to thoracic endometriosis. Intern. Med..

[24] Kovarik J.L., Toll G.D. (1966). Thoracic endometriosis with recurrent spontaneous pneumothorax. JAMA.

[25] Aissa S., Benzarti W., Alimi F., Gargouri I., Salem H.B., Aissa A., Fathallah K., Abdelkade A.B., Alouini R., Garrouche A. (2017). Catamenial pneumothorax revealing diaphragmatic endometriosis: A case report and revue of literature. Pan Afr. Med. J..

[26] Devue K., Coenye K., Verhaeghe W. (2005). A case of catamenial pneumothorax caused by thoracic endometriosis. Eur. J. Emerg. Med..

[27] Cieslik L., Haider S.S., Fisal L., Rahmaan J.A.A., Sachithanandan A. (2013). Minimally invasive thoracoscopic mesh repair of diaphragmatic fenestrations for catamenial pneumothorax due to likely thoracic endometriosis: A case report. Med. J. Malays..

[28] Guenther T.M., Gustafson J.D., Pribyl S.M., Wozniak C.J. (2020). Recurrent spontaneous pneumothorax in a 47-year-old woman. Mil. Med..

[29] Shiraishi T. (1991). Catamenial pneumothorax: Report of a case and review of the Japanese and non-Japanese literature. Thorac. Cardiovasc. Surg..

[30] Korom S., Canyurt H., Missbach A., Schneiter D., Kurrer M.O., Haller U., Keller P.J., Furrer M., Weder W. (2004). Catamenial pneumothorax revisited: Clinical approach and systematic review of the literature. J. Thorac. Cardiovasc. Surg..

[31] Ichiki Y., Nagashima A., Yasuda M., Takenoyama M., Toyoshima S. (2015). Surgical treatment of catamenial pneumothorax: Report of three cases. Asian J. Surg..

[32] Visouli A.N., Darwiche K., Mpakas A., Zarogoulidis P., Papagiannis A., Tsakiridis K., Machairiotis N., Stylianaki A., Katsikogiannis N., Courcoutsakis N. (2012). Catamenial pneumothorax: A rare entity? Report of 5 cases and review of the literature. J. Thorac. Dis..

[33] Kardaman N., Nizami M., Marciniak S., Hogan J., Aresu G. (2022). Catamenial pneumothorax. Ann. R. Coll. Surg. Engl..

[34] Koike S., Kobayashi N., Miyazawa M. (2023). Positive outcome of diaphragm covering and total pleural covering techniques for catamenial pneumothorax. J. Surg. Case. Rep..

[35] Duyos I., López-Carrasco A., Hernández A., Zapardiel I., De Santiago J. (2014). Management of thoracic endometriosis: Single institution experience. Eur. J. Obstet. Gynecol. Reprod. Biol..

[36] Poh C.L., Yan T.D., Vallely M.P., Bannon P.G., Mccaughan B.C. (2011). Pulmonary parenchymal endometriosis presenting as bilateral pneumothoraces. J. Obstet. Gynaecol..

[37] Fang H.Y., Jan C.I., Chen C.K., Chen W.T. (2012). Catamenial pneumothorax due to bilateral pulmonary endometriosis. Respir. Care.

[38] Low Q.J., Cheo S.W., Wong W.H., Goh K.S. (2019). Endometriosis—A rare cause of primary spontaneous pneumothorax. Med. J. Malays..

[39] Papafragaki D., Concannon L. (2008). Catamenial pneumothorax: A case report and review of the literature. J. Womens Health.

[40] Oger P., Alifano M., Regnard J.F., Gompel A. (2006). Difficult management of recurrent catamenial pneumothorax. Gynecol. Endocrinol..

[41] Azizad-Pinto P., Clarke D. (2014). Thoracic endometriosis syndrome: Case report and review of the literature. Perm. J..

[42] Lua L.L., Tran K., Desai J. (2017). Refractory thoracic endometriosis syndrome with bilateral hemothorax. J. Obstet. Gynaecol. Res..

[43] Nemeş R.M., Paleru C., Dănăilă O., Ianoşi E.S., Pop C.S., DiŢescu D., Streba C.T., NiŢu M.F. (2015). Thoracic endometriosis with a long delay in diagnosis. Rom. J. Morphol. Embryol..

[44] Ziedalski T.M., Sankaranarayanan V., Chitkara R.K. (2004). Thoracic endometriosis: A case report and literature review. J. Thorac. Cardiovasc. Surg..

[45] McCann M.R., Schenk W.B., Nassar A., Maimone S. (2020). Thoracic endometriosis presenting as a catamenial hemothorax with discordant video-assisted thoracoscopic surgery. Radiol. Case Rep..

[46] Dhanaworavibul K., Hanprasertpong J., Cheewadhanaraks S., Buhachat R. (2006). Bilateral pleural endometriosis. J. Obstet. Gynaecol. Res..

[47] Ismail Y., Kamaruzzaman A. (2004). Thoracic endometriosis: A report of two cases. Med. J. Malays..

[48] Junejo S.Z., Lubana S.S., Shina S.S., Tuli S.S. (2018). A case of thoracic endometriosis syndrome presenting with recurrent catamenial pneumothorax. Am. J. Case Rep..

[49] Aguilar-Shea A.L., Gallardo-Mayo C. (2012). Thoracic endometriosis as cause of recurrent pneumothorax. QJM Int. J. Med..

[50] Tsunezuka Y., Sato H., Kodama T., Shimizu H., Kurumaya H. (2012). Expression of CA125 in thoracic endometriosis in a patient with catamenial pneumothorax. Respiration.

[51] Inam H., Inam S., Tahir M. (2016). Catamenial pneumothorax: A case report. J. Pak. Med. Assoc..

[52] Baisi A., Raveglia F., Simone M.D., Calati A.M., Leporati A., Cioffi U. (2020). Endometriosis-related pneumothorax after in vitro fertilization embryo transfer procedure: A case report. J. Thorac. Cardiovasc. Surg..

[53] Alifano M., Cancellieri A., Fornelli A., Trisolini R., Boaron M. (2004). Endometriosis-related pneumothorax: Clinicopathologic observations from a newly diagnosed case. J. Thorac. Cardiovasc. Surg..

[54] Yamazaki S., Ogawa J., Koide S., Shohzu A., Osamura Y. (1980). Catamenial pneumothorax associated with endometriosis of the diaphragm. Chest.

[55] Garg V., McKenzie Gray B. (2008). An unusual case of catamenial pneumothorax. J. Obstet. Gynaecol..

[56] Maurer E.R., Schaal J.A., Mendez F.L. (1958). Chronic recurring spontaneous pneumothorax due to endometriosis of the diaphragm. J. Am. Med. Assoc..

[57] Bagan P., Le Pimpec Barthes F., Assouad J., Souilamas R., Riquet M. (2003). Catamenial pneumothorax: Retrospective study of surgical treatment. Ann. Thorac. Surg..

[58] Grunewald R.A., Wiggins J. (1988). Pulmonary endometriosis mimicking an acute abdomen. Postgrad. Med. J..

[59] Davies R. (1968). Recurring spontaneous pneumothorax concomitant with menstruation. Thorax.

[60] Poyraz A.S., Kilic D., Hatipoglu A., Demirhan B.A. (2005). A very rare entity: Catamenial pneumothorax. Asian Cardiovasc. Thorac. Ann..

[61] Mukku V.K., Cassidy E., Negulescu C., Jagneaux T., Godke J. (2019). Large spontaneous right catamenial pneumothorax with diaphragmatic defect and liver herniation. Case Rep. Pulmonol..

[62] Uemura T., Matsuyama A., Minaguchi H., Ikeda H. (1958). Danazol (an antigonadotropin) in the treatment of catamenial pneumothorax. Asia Oceania J. Obstet. Gynaecol..

[63] Sharma N., Todhe P., Ochieng P., Ramakrishna S. (2020). Refractory thoracic endometriosis. BMJ Case Rep..

[64] El Haj Chehade A., Nasir A.B., Peterson J.E.G., Ramseyer T., Bhardwaj H. (2022). Thoracic endometriosis presenting as hemopneumothorax. Monaldi Arch. Chest. Dis..

[65] Dong B., Wu C.L., Sheng Y.L., Wu B., Ye G.C., Liu Y.F., Li S.H., Han L., Qi Y. (2021). Catamenial pneumothorax with bubbling up on the diaphragmatic defects: A case report. BMC Womens Health.

[66] Miedziarek C., Kasprzyk M. (2022). Catamenial pneumothorax—Are there benefits of cooperation between the surgeon and the gynaecologist?. Prz. Menopauzalny.

[67] Gupta V., Noh K.W., Maschek H., Thal S., Welter S. (2022). A unique case of thoracic endometriosis syndrome and pulmonary Langerhans’ cell histiocytosis: Six recurrent pneumothoraces. Respir. Med. Case Rep..

[68] Mikroulis D.A., Didilis V.N., Konstantinou F., Vretzakis G.H., Bougioukas G.I. (2008). Catamenial pneumothorax. Thorac. Cardiovasc. Surg..

[69] Solanki K.K., Shook M., Yorke J., Vanlandingham A. (2023). A rare case of catamenial pneumothorax and a review of the current literature. Cureus.

[70] Pratomo I.P., Putra M.A., Bangun L.G., Soetartio I.M., Maharani M.A.P., Febriana I.S., Soehardiman D., Prasenohadi P., Kinasih T. (2023). Video-assisted surgical diagnosis and pleural adhesion management in catamenial pneumothorax: A case and literature review. Respirol. Case Rep..

[71] Das Posses Bridi G., de Oliveira M.R., Carvalho C.R.R., do Nascimento E.C.T., Baldi B.G. (2024). Thoracic endometriosis presenting as diffuse cystic lung disease: A rare case report. Pulmonology.

[72] Maniglio P., Ricciardi E., Meli F., Vitale S.G., Noventa M., Vitagliano A., Valenti G., La Rosa V.L., Laganà A.S., Caserta D. (2017). Catamenial pneumothorax caused by thoracic endometriosis. Radiol. Case Rep..

[73] Matalliotakis I.M., Goumenou A.G., Koumantakis G.E., Neonaki M.A., Koumantakis E.E., Arici A. (2002). Pulmonary endometriosis in a patient with unicornuate uterus and noncommunicating rudimentary horn. Fertil. Steril..

[74] Zhu C.R., Suen M.W.H., Gilbert S., Singh S.S. (2019). Laparoscopic management of diaphragmatic endometriosis. J. Obstet. Gynaecol. Can..

[75] Gaichies L., Blouet M., Comoz F., Foulon A., Heyndrickx M., Fauvet R. (2019). Non-traumatic diaphragmatic rupture with liver herniation due to endometriosis: A rare evolution of the disease requiring multidisciplinary management. J. Gynecol. Obstet. Hum. Reprod..

[76] Blanco S., Hernando F., Gómez A., González M.J., Torres A.J., Balibrea J.L. (1998). Catamenial pneumothorax caused by diaphragmatic endometriosis. J. Thorac. Cardiovasc. Surg..

[77] Picozzi G., Beccani D., Innocenti F., Grazzini M., Mascalchi M. (2007). MRI features of pleural endometriosis after catamenial haemothorax. Thorax.

[78] Gilabert-Estelles J., Zorio E., Castello J.M., Estelles A., Gilabert-Aguilar J. (2020). Laparoscopic approach to right diaphragmatic endometriosis with argon laser: Case report. J. Minim. Invasive Gynecol..

[79] Jacob A., Coker A., Stamenkovic S.A. (2023). Robotic-assisted thoracic surgery approach to thoracic endometriosis syndrome with unilateral diaphragmatic palsy. Case. Rep. Surg..

[80] Trehan K., Mungo B., Molena D. (2015). Recurrent thoracic endometriosis with extensive adhesions after talc pleurodesis. Surgery.

[81] Morita Y., Tsutsumi O., Taketani Y. (1997). Successful hormonal treatment of pulmonary parenchymal endometriosis. Int. J. Gynecol. Obstet..

[82] Ezemba N., Okafor O.C., Emeruem N.U., Adiri C.O. (2021). Thoracic endometriosis syndrome in Nigeria: A single-centre experience. Interact. Cardiovasc. Thorac. Surg..

[83] Elliot D.L., Barker A.F., Dixon L.M. (1958). Catamenial hemoptysis. Chest.

[84] Keating E., Lawson R.A., Li T.C., Makris M. (2000). Monthly haemoptysis in a woman with platelet storage pool disease. Clin. Lab. Haematol..

[85] Leonardo-Pinto J.P., Benetti-Pinto C.L., Quagliato I., Yela D.A. (2018). Hemoptysis and endometriosis: An unusual association—Case report and review of the literature. Rev. Bras. Ginecol. Obstet..

[86] Marshall M.B., Ahmed Z., Kucharczuk J.C., Kaiser L.R., Shrager J.B. (2005). Catamenial pneumothorax: Optimal hormonal and surgical management. Eur. J. Cardiothorac. Surg..

[87] Ogunkoya J.O., Solaja T.O., Ogunlade A.F., Ogunmola M.I. (2022). Thoracic endometriosis: A presentation of an uncommon disease in a black African woman. Case Rep. Med..

[88] Sturney S.C., Meecham-Jones J., White A. (2013). An uncommon case of hydropneumothorax and haemoptysis. BMJ Case Rep..

[89] Kervancioglu S., Andic C., Bayram N., Telli C., Sarica A., Sirikci A. (2008). Bronchial artery embolization in the management of pulmonary parenchymal endometriosis with hemoptysis. Cardiovasc. Interv. Radiol..

[90] Nezhat C., Main J., Paka C., Nezhat A., Beygui R.E. (2014). Multidisciplinary treatment for thoracic and abdominopelvic endometriosis. J. Soc. Laparosc. Robot. Surg..

[91] Rawala M.S., Khaliq M.F., Iqbal M.A., Naqvi S.T.S., Farhan K., Myers A., Helmick K. (2018). A rare case of cyclical hemothorax: Thoracic endometriosis syndrome. Case Rep. Pulmonol..

[92] Adesanya O.A., Kolawole O.E. (2020). Thoracic endometriosis syndrome: Cutting the gordian knot—A case report and review of the literature. Int. J. Surg. Case Rep..

[93] Rychlik D.F., Bieber E.J. (2001). Thoracic endometriosis syndrome resembling pulmonary embolism. J. Am. Assoc. Gynecol. Laparosc..

[94] Byanyima R.K. (2001). Menstruation in an unsual place: A case of thoracic endometriosis in Kampala, Uganda. Afr. Health Sci..

[95] Moffatt S.D., Mitchell J.D. (2002). Massive pleural endometriosis. Eur. J. Cardiothorac. Surg..

[96] Nizami M., Mani A., Begum S. (2019). Catamenial hemothorax: A rare case of thoracic endometriosis. Ann. Thorac. Surg..

[97] Dogra N., Luthra A., Chauhan R., Bajaj R., Gourav K.P. (2020). Recurrent unilateral hemorrhagic pleural effusion: A rare manifestation of thoracic endometriosis syndrome. Ann. Card. Anaesth..

[98] Kyo S., Takakura M., Nishida S., Ozaki S., Oda M., Inoue M. (2012). Massive hemothorax due to diaphragmatic endometriosis after a laparoscopic cystectomy of an ovarian endometrioma in a patient without a history of thoracic endometriosis. Arch. Gynecol. Obstet..

[99] Somani A., Pillai S., Maryam M., Chakrapani A. (2020). A rare massive presentation of catamenial hemothorax. Am. J. Emerg. Med..

[100] Davis B.M., Goldstraw E., Bhowmik A., José R.J. (2016). A gynaecological cause of spontaneous haemopneumothorax. Br. J. Hosp. Med..

[101] Arakawa S., Matsudaira H., Noda Y., Yamashita M., Hirano J., Ogawa M., Ohtsuka T. (2021). Catamenial pneumothorax with partial liver herniation due to diaphragmatic laceration: A case report and literature review. J. Cardiothorac. Surg..

[102] Yildirim M., Oztekin O., Oztekin D. (2011). Recurrent chest pain, as a presenting sign of ovarian endometrioma. ISRN Surg..

[103] Chetambath R., Kumar P., Nandini V., Chandran S., Chacko A. (2023). Catamenial haemothorax—A rare cause of pleural effusion. Lung India.

[104] Zippl A.L., Yang Mohsin W.S., Gasser E., Henninger B., Widschwendter A., Kafka R., Seeber B. (2022). Phrenic nerve paralysis after bipolar electrocoagulation of endometriosis of the diaphragm: Case report and mini review. F&S Rep..

[105] Ribeiro M., Freire T., Leite F., Werebe E., Cabrera Carranco R., Kondo William W. (2021). The importance of early diagnosis and treatment of incidental tension pneumothorax during robotic assisted laparoscopy for diaphragmatic endometriosis: A report of two cases. Facts Views Vis. Obgyn.

[106] Ciriaco P., Negri G., Libretti L., Carretta A., Melloni G., Casiraghi M., Bandiera A., Zannini P. (2009). Surgical treatment of catamenial pneumothorax: A single centre experience. Interact. Cardiovasc. Thorac. Surg..

[107] Ottolina J., De Stefano F., Viganò P., Ciriaco P., Zannini P., Candiani M. (2017). Thoracic endometriosis syndrome: Association with pelvic endometriosis and fertility status. J. Minim. Invasive Gynecol..

[108] Kawaguchi Y., Fujita T., Hanaoka J. (2015). Catamenial pneumothorax with bullae. Ann. Thorac. Surg..

[109] Singh M., Singh R.B., Singh A.B., Carballo A.L., Jain A. (2021). Thoracic endometriosis: Still a diagnostic dilemma. Cureus.

[110] Rangunwala J., Sitta J., Vyas K., Roda M. (2020). Multimodality thoracoabdominal imaging findings in a rare case of thoracic endometriosis syndrome. Cureus.

[111] Di Palo S., Mari G., Castoldi R., Staudacher C., Taccagni G., Di Carlo V. (1989). Endometriosis of the lung. Respir. Med..

[112] Kim Y.D., Min K.O., Moon S.W. (2020). Thoracoscopic treatment of recurrent pneumothorax in a pregnant woman: A case of ectopic deciduosis. Thorac. Cardiovasc. Surg..

[113] Rousset-Jablonski C., Alifano M., Plu-Bureau G., Camilleri-Broet S., Rousset P., Regnard J.F., Regnard J.F., Gompel A. (2011). Catamenial pneumothorax and endometriosis-related pneumothorax: Clinical features and risk factors. Hum. Reprod..

[114] Marques V.D., de Mattos L.A., Pimenta A.M., Pelloso S.M., Bandeira C.O.P., Lemos M.M., Moraes W.A.S., Carvalho M.D.B. (2020). Resection of pulmonary endometriosis by video-assisted thoracoscopic surgery using bronchoscopy as a preoperative strategy. Ann. Thorac. Surg..

[115] Kiyan E., Kilicaslan Z., Caglar E., Yilmazbayhan D., Tabak L., Gürgan M. (2002). An unusual radiographic finding in pulmonary parenchymal endometriosis. Acta. Radiol..

[116] Nezhat C., King L.P., Paka C., Odegaard J., Beygui R. (2012). Bilateral thoracic endometriosis affecting the lung and diaphragm. J. Soc. Laparosc. Robot. Surg..

[117] Yu J.H., Lin X.Y., Wang L., Liu Y., Fan C.F., Zhang Y., Wang E.H. (2013). Endobronchial endometriosis presenting as central-type lung cancer: A case report. Diagn. Pathol..

[118] Alifano M., Roth T., Broët S.C., Schussler O., Magdeleinat P., Regnard J.F. (2003). Catamenial pneumothorax: A prospective study. Chest.

[119] Ravindran P., Raj R.J., Parameswaran K. (1993). Concurrent catamenial hemothorax and hemopneumothorax. Chest.

[120] Black H., Sigal D., Barnes D., Felisky C., Follette D., Harper R. (2005). A 25-year-old patient with spontaneous hemothorax. Chest.

[121] Kyejo W., Ismail A., Rubagumya D., Bakari R., Kaguta M., Matillya N. (2022). Shortness of breath in a young lady, rare case report of thoracic endometriosis. Int. J. Surg. Case Rep..

[122] Keijzer S., Oosterhuis W., Hazelbag H.M., Meuleman T. (2021). Pathological diagnosis of thoracic endometriosis. BMJ Case Rep..

[123] Kalbi D.P., Al Sbihi A.F., Manasrah N., Chaudhary A.J., Iqbal S. (2021). A thoracic endometriosis-related catamenial hemopneumothorax in a woman with premature ovarian failure. Cureus.

[124] Mittal A., Jomaa D., Hassan Z., Hines J., Thavarajah K. (2022). Catamenial pneumothorax in the setting of a recent stroke. Cureus.

[125] Chittemsetti S., Baikunje N., Hosmane G.B., Bhat S. (2021). Recurrent pleural effusion secondary to endometriosis: A rare malady. BMJ Case Rep..

[126] Patrucco Reyes S., Amoah K., Rahi M.S., Gunasekaran K. (2021). A case of hemothorax as manifestation of thoracic endometrial syndrome. J. Investig. Med. High Impact Case Rep..

[127] Viswanath V., Khurana A., Goyal A., Niwariya Y., Singh M.P., Panwar H., Goel G. (2018). Thoracic endometriosis presenting with bilateral hydropneumothorax. Sultan Qaboos Univ. Med. J..

[128] Bahall V., De Barry L., Singh K. (2022). Thoracic endometriosis masquerading as Meigs’ syndrome in a young woman: A case report and literature review. Case Rep. Womens Health.

[129] Ozvaran M.K., Baran R., Soğukpmar O., Uzman O., Sahin K., Kocadelioglu I., Aksoy F., Altun S. (2006). Histopathological diagnosis of endobronchial endometriosis treated with argon laser. Respirology.

[130] Kuo P.H., Wang H.C., Liaw Y.S., Kuo S.H. (1996). Bronchoscopic and angiographic findings in tracheobronchial endometriosis. Thorax.

[131] Cutz J.C., Woods J.S., Mitchell J.H., Colby T.V., Leslie K.O. (2007). A common presentation with a rare cause. Eur. Respir. J..

[132] Cassina P.C., Weder W., Hauser M., Kacl G., Imthurn B., Schröder S. (1997). Catamenial hemoptysis. Chest.

[133] 1Sanada T., Park J., Hagiwara M., Ikeda N., Nagai T., Matsubayashi J., Saito K. (2018). CT and MRI findings of bronchopulmonary endometriosis: A case presentation. Acta. Radiol. Open.

[134] Derman A.Y., Sperling D., Merav A., Jain V.R., Levin M., Jana S., Haramati L.B. (2007). Endometrioma presenting as a cavitary lung mass with intense 18F-FDG uptake on PET-CT. J. Thorac. Imaging.

[135] Mulette P., Jacquet A., Durlach A., Papathanassiou D., Lalire P., Graesslin O., Delepine G., Dury S., Dormoy V., Perotin J.M. (2021). Pulmonary cavitations with increased 18F-FDG uptake revealing a thoracic endometriosis. Medicine.

[136] Bateman E.D., Morrison S.C. (1990). Catamenial haemoptysis from endobronchial endometriosis—A case report and review of previously reported cases. Respir. Med..

[137] Orriols R., Muñoz X., Álvarez A., Sampol G. (1998). Chest CT scanning: Utility in lung endometriosis. Respir. Med..

[138] Inoue T., Kurokawa Y., Kaiwa Y., Abo M., Takayama T., Ansai M., Satomi S. (2001). Video-assisted thoracoscopic surgery for catamenial hemoptysis. Chest.

[139] Weber F. (2001). Catamenial hemoptysis. Ann. Thorac. Surg..

[140] Jitruckthai P. (2015). A woman with recurrent hemoptysis, a rare etiology. J. Med. Assoc. Thai..

[141] Kristiansen K., Fjeld N.B. (1993). Pulmonary endometriosis causing haemoptysis: Report of a case treated with lobectomy. Scand. J. Thorac. Cardiovasc. Surg..

[142] Hope-Gill B., Prathibha B.V. (2003). Catamenial haemoptysis and clomiphene citrate therapy. Thorax.

[143] Park Y.B., Heo G.M., Moon H.K., Cho S.J., Shin Y.C., Eom K.S., Kim C.H., Lee J.Y., Mo E.K., Jung K.S. (2006). Pulmonary endometriosis resected by video-assisted thoracoscopic surgery. Respirology.

[144] Lawrence H.C. (1988). Pulmonary endometriosis in pregnancy. Am. J. Obstet. Gynecol..

[145] Shiota Y., Umemura S., Arikita H., Horita N., Hiyama J., Tetsuya Ono T., Sasaki N., Taniyama K., Yamakido M. (2001). A case of parenchymal pulmonary endometriosis, diagnosed by cytologic examination of bronchial washing. Respiration.

[146] Hachiya T., Okada M., Takamizawa A., Hasegawa M., Honda T., Kubo K. (2003). Catamenial hemoptysis. Intern. Med..

[147] Ryu J.S., Song E.S., Lee K.H., Cho J.H., Kwak S.M., Lee H.L. (2007). Natural history and therapeutic implications of patients with catamenial hemoptysis. Respir. Med..

[148] Terada Y., Chen F., Shoji T., Itoh H., Wada H., Hitomi S. (1999). A case of endobronchial endometriosis treated by subsegmentectomy. Chest.

[149] Assor D. (1972). Endometriosis of the lung: Report of a case. Am. J. Clin. Pathol..

[150] Fujimoto K., Kasai H., Suga M., Sugiura T., Terada J., Suzuki H., Oota M., Yoshino I., Nakatani Y., Tatsumi K. (2017). Pulmonary endometriosis which probably occurred through hematogenous metastasis after artificial abortion. Intern. Med..

[151] Katoh O., Yamada H., Aoki Y., Matsumoto S., Kudo S. (1990). Utility of angiograms in patients with catamenial hemoptysis. Chest.

[152] Puma F., Carloni A., Casucci G., Puligheddu C., Urbani M., Porcaro G. (2003). Successful endoscopic Nd-YAG laser treatment of endobronchial endometriosis. Chest.

[153] Jang H.I., Kim S.E., Kim T.J., Lee Y.Y., Choi C.H., Lee J.W., Kim B.G., Bae D.S. (2017). Catamenial hemoptysis accompanied by subcutaneous endometriosis treated with combination therapy. Obstet. Gynecol. Sci..

[154] Matsushima K., Ono M., Hayashi S., Sonoda D., Matsui Y., Shiomi K., Satoh Y., Ohbu M. (2020). Resection of intra-pulmonary endometriosis by video-assisted thoracoscopic surgery under pre-operative CT-guided marking synchronized with menstrual cycle. Gen. Thorac. Cardiovasc. Surg..

[155] Lindenberg K., Schmid J., Rüttner J., Sulser H., Schmid M. (1975). Endometriosis of the lung: Case report. Arch. Gynakol..

[156] Zanetti G., Hochhegger B., Marchiori E. (2020). Pulmonary endometriosis: An unusual cause of hemoptysis. J. Bras. Pneumol..

[157] Tong S.S., Yin X.Y., Hu S.S., Cui Y., Li H.T. (2019). Case report of pulmonary endometriosis and review of the literature. J. Int. Med. Res..

[158] Bala A., Salim R.A., Deliwala S., Obeid M., Bachuwa G. (2020). Cyclical hemoptysis and pelvic pain in a young female: A sign of thoracic endometriosis syndrome. Cureus.

[159] Yisa S.B., Okenwa A.A., Husemeyer R.P. (2004). Treatment of endometriotic catamenial haemoptysis with etonogestrel subdermal implant. BJOG.

[160] Yao J., Zheng H., Nie H., Li C.F., Zhang W., Wang J.J. (2023). Endometriosis of the lung: A case report and review of literature. World J. Clin. Cases.

[161] Verhulst E., Bafort C., Tomassetti C., Wolthuis A., Bielen D., Coolen J., Weynand B., Platteeuw L., Meuleman C., Van Raemdonck D. (2022). Endometriotic lung cyst causing catamenial hemoptysis; A case report and review of literature. Acta. Chir. Belg..

[162] Son J.H., Kim D.H., Park J.M., Lee S.K. (2021). Successful treatment of catamenial hemoptysis by single-incision thoracoscopic left S9+10 segmentectomy using indocyanine green injection-assisted targeting. Gen. Thorac. Cardiovasc. Surg..

[163] Chen C.L., Huang W.C., Cheng W.C. (2020). Catamenial hemoptysis. QJM Int. J. Med..

[164] Chen C., Zhai K., Tang Y., Qu W., Zuo J., Ke X., Song Y. (2021). Thoracic endometriosis: A case report and review of the literature. Ann. Palliat. Med..

[165] Chen M.L., Li C.Y. (2021). Thoracic endometriosis. N. Engl. J. Med..

[166] Aboujaoude N., Iskandar M., Tannouri F. (2021). Catamenial hemoptysis: A case report of pulmonary endometriosis. Eur. J. Radiol. Open.

[167] Ganesan P.R., Kang D., Khan Z., Milteer H.B. (2023). Diaphragmatic endometriosis presenting as recurrent catamenial pneumothorax: A case report. Cureus.

[168] Bozkanat K.M., West N.E., Ladores S., Montemayor K., Tupayachi Ortiz M.G., Christianson M., Jain R. (2021). Catamenial haemoptysis in females with cystic fibrosis: A case series with review of management strategies. Respirol. Case Rep..

[169] Chao Y.K., Ko P.J., Yeow K.M., Liu Y.H. (2006). Video-assisted thoracoscopic surgery for catamenial hemoptysis: The rationale of preoperative computed tomography-guided hook-wire localization. Surg. Laparosc. Endosc. Percutaneous Tech..

[170] Fleishman S., Davidson J.F. (1959). Vicarious menstruation, a likely case of pulmonary endometriosis. Lancet.

[171] Rodman M.H., Jones C.W. (1962). Catamenial hemoptysis due to bronchial endometriosis. N. Engl. J. Med..

[172] Hertzanu Y., Heimer D., Hirsch M. (1987). Computed tomography of pulmonary endometriosis. Comput. Radiol..

[173] Lu M.S., Liu Y.H., Wu Y.C., Hsieh M.J., Liu H.P. (2006). What we see is not what we get in catamenial haemoptysis: Catamenial haemoptysis. Int. J. Clin. Pract..

[174] Haruki T., Fujioka S., Adachi Y., Miwa K., Taniguchi Y., Nakamura H. (2007). Successful video-assisted thoracic surgery for pulmonary endometriosis: Report of a case. Surg. Today.

[175] Lee C.H., Huang Y.C., Huang S.F., Wu Y.K., Kuo K.T. (2009). Thoracic endometriosis: Rare presentation as a solitary pulmonary nodule with eccentric cavitations. Thorax.

[176] Nakashima Y., Shoji F., Osoegawa A., Yoshino I., Maehara Y. (2011). Catamenial hemoptysis treated by video-assisted thoracic surgery: Report of a case. Surg. Today.

[177] Chatra P.S. (2012). Thoracic endometriosis: A case report. J. Radiol. Case Rep..

[178] Okur A., Metin B., İntepe Y.S., Serin H.İ. (2014). Catamenial hemoptysis: A case report. Tuberk. Toraks..

[179] Furuya K., Otsuka H., Koezuka S., Makino T., Hata Y., Wakayama M., Shibuya K., Iyoda A. (2017). Resection of pulmonary endometriosis using video-assisted thoracoscopic surgery under preoperative CT-guided marking. Gen. Thorac. Cardiovasc. Surg..

[180] Alwadhi S., Kohli S., Chaudhary B., Gehlot K. (2016). Thoracic endometriosis—A rare cause of haemoptysis. J. Clin. Diagn. Res..

[181] Koizumi T., Inagaki H., Takabayashi Y., Kubo K. (1999). Successful use of gonadotropin-releasing hormone agonist in a patient with pulmonary endometriosis. Respiration.

[182] Takahashi M., Matsukura T., Hirai T., Mino N. (2013). Recurrent catamenial hemopneumothorax treated by coverage with polyglycolic acid sheets. J. Thorac. Cardiovasc. Surg..

[183] Nezhat C., Nicoll L.M., Bhagan L., Huang J.Q., Bosev D., Hajhosseini B., Beygui R.E. (2009). Endometriosis of the diaphragm: Four cases treated with a combination of laparoscopy and thoracoscopy. J. Minim. Invasive Gynecol..

[184] Huber M., Wierrani F., Böhm G., Hauck H., Lintner F., Grünberger W. (1999). Multiple endometrial stromal nodules with sparse cysts and glands in the lung—A nodular variation of endometriosis that may mimic metastases of sarcoma. Pathol. Res. Pract..

[185] Ludwig M., Bauer O., Wiedemann G.J., Diedrich K. (2001). Ureteric and pulmonary endometriosis. Arch. Gynecol. Obstet..

[186] Brown A., Deshmukh M., Tavare A., Gillmore R. (2018). Endometriosis: A rare cause of multiple lung nodules on imaging. Br. J. Hosp. Med..

[187] Choi S.Y., Kim C.K., Park C.B. (2013). Successful treatment of catamenial hemoptysis by video-assisted thoracoscopic surgery. Thorac. Cardiovasc. Surg..

[188] Kim C.J., Nam H.S., Lee C.Y., Yum H.K., Yang S.H., Seo K.H., Son C.H., Kim D.J., Jang S.H., Chung M.P. (2010). Catamenial hemoptysis: A nationwide analysis in Korea. Respiration.

[189] Legras A., Mansuet-Lupo A., Rousset-Jablonski C., Bobbio A., Magdeleinat P., Roche N., Regnard J.F., Gompel A., Damotte D., Alifano M. (2014). Pneumothorax in women of child-bearing age: An update classification based on clinical and pathologic findings. Chest.

[190] Attaran S., Bille A., Karenovics W., Lang-Lazdunski L. (2013). Videothoracoscopic repair of diaphragm and pleurectomy/abrasion in patients with catamenial pneumothorax: A 9-year experience. Chest.

[191] Saito T., Saito Y., Fukumoto K.J., Matsui H., Nakano T., Taniguchi Y., Kaneda H., Konobu T., Tsuta K., Murakawa T. (2018). Clinical and pathological characteristics of spontaneous pneumothorax in women: A 25-year single-institutional experience. Gen. Thorac. Cardiovasc. Surg..

[192] Hwang S.M., Lee C.W., Lee B.S., Park J.H. (2015). Clinical features of thoracic endometriosis: A single center analysis. Obstet. Gynecol. Sci..

[193] Cameron H.M., Path M.C., Park W.W., Path M.C. (1965). Decidual tissue within the lung. BJOG.

[194] Wang H.C., Kuo P.H., Kuo S.H., Luh K.T. (2000). Catamenial hemoptysis from tracheobronchial endometriosis: Reappraisal of diagnostic value of bronchoscopy and bronchial brush cytology. Chest.

[195] Chung S.Y., Kim S.J., Kim T.H., Ryu W.G., Park S.J., Lee D.Y., Paik H.C., Kim H.J., Cho S.H., Kim J.K. (2005). Computed tomography findings of pathologically confirmed pulmonary parenchymal endometriosis. J. Comput. Assist. Tomogr..

[196] Kim C.H., Lee D.Y., Moon D.H., Lee S. (2021). Percutaneous cryoablation of multiple pulmonary endometriosis. J. Chest Surg..

[197] Huang H., Li C., Zarogoulidis P., Darwiche K., Machairiotis N., Yang L., Simoff M., Celis E., Zhao T., Zarogoulidis K. (2013). Endometriosis of the lung: Report of a case and literature review. Eur. J. Med. Res..

[198] Hong Y.J., Paik H.C., Kim H.J., Lee D.Y., Kim S.J., Cho S.H., Oh Y.M. (1999). A case of parenchymal pulmonary endometriosis. Yonsei Med. J..

[199] Kumakiri J., Kumakiri Y., Miyamoto H., Kikuchi I., Arakawa A., Kitade M., Takeda S. (2010). Gynecologic evaluation of catamenial pneumothorax associated with endometriosis. J. Minim. Invasive Gynecol..

[200] Muramatsu T., Shimamura M., Furuichi M., Nishii T., Ishimoto S., Morooka H., Yagasaki C., Ohmori K., Shiono M. (2010). Surgical treatment of catamenial pneumothorax. Asian J. Surg..

[201] Lopes S., Maciel J., Cabral Bastos P., Pinho P. (2017). What about having a hydropneumothorax every month?. Rev. Port. Cir. Cardiotorac. Vasc..

[202] Rometti M., Patti L. (2023). Catamenial pneumothorax in a patient with endometriosis: A case report. Cureus.

[203] Guidry G.G., George R.B., Payne D.K. (1990). Catamenial hemoptysis: A case report and review of the literature. J. La State Med. Soc..

[204] Joseph J., Reed C.E., Sahn S.A. (1994). Thoracic endometriosis. Recurrence following hysterectomy with bilateral salpingo-oophorectomy and successful treatment with talc pleurodesis. Chest.

[205] Kirschner P.A. (1998). Porous diaphragm syndromes. Chest Surg. Clin. N. Am..

[206] Yeh T.J. (1967). Endometriosis within the thorax: Metaplasia, implantation, or metastasis?. J. Thorac. Cardiovasc. Surg..

[207] Olive D.L., Pritts E.A. (2001). Treatment of endometriosis. N. Engl. J. Med..

[208] Alifano M., Trisolini R., Cancellieri A., Regnard J.F. (2006). Thoracic endometriosis: Current knowledge. Ann. Thorac. Surg..

[209] Schwartz K., Llarena N.C., Rehmer J.M., Richards E.G., Falcone T. (2020). The role of pharmacotherapy in the treatment of endometriosis across the lifespan. Expert Opin. Pharmacother..

